# Optimizing Deep Learning-Based Crack Detection Using No-Reference Image Quality Assessment in a Mobile Tunnel Scanning System

**DOI:** 10.3390/s25175437

**Published:** 2025-09-02

**Authors:** Chulhee Lee, Donggyou Kim, Dongku Kim

**Affiliations:** Department of Geotechnical Engineering Research, Korea Institute of Civil Engineering and Building Technology (KICT), Goyang-Si 10223, Gyeonggi-Do, Republic of Korea; lch@kict.re.kr (C.L.);

**Keywords:** mobile tunnel scanning system, convolutional neural network, crack detection, no-reference image quality assessment, motion blur

## Abstract

The mobile tunnel scanning system (MTSS) enables efficient tunnel inspection; however, motion blur (MB) generated at high travel speeds remains a major factor undermining the reliability of deep-learning-based crack detection. This study focuses on investigating how horizontally oriented MB in MTSS imagery affects the crack-detection performance of convolutional neural networks (CNNs) and proposes a data-centric quality-assurance framework that leverages no-reference image quality assessment (NR-IQA) to optimize model performance. By intentionally applying MB to both public and real-world MTSS datasets, we analyzed performance changes in ResNet-, VGG-, and AlexNet-based models and established the correlations between four NR-IQA metrics (BRISQUE, NIQE, PIQE, and CPBD) and performance (F1 score). As the MB intensity increased, the F1 score of ResNet34 dropped from 89.43% to 4.45%, confirming the decisive influence of image quality. PIQE and CPBD exhibited strong correlations with F1 (−0.87 and +0.82, respectively), emerging as the most suitable indicators for horizontal MB. Using thresholds of PIQE ≤ 20 and CPBD ≥ 0.8 to filter low-quality images improved the AlexNet F1 score by 1.46%, validating the effectiveness of the proposed methodology. The proposed framework objectively assesses MTSS data quality and optimizes deep learning performance, enhancing the reliability of intelligent infrastructure maintenance systems.

## 1. Introduction

Cracks in tunnel concrete linings are indicators of tunnel safety and durability, and their accurate detection is crucial for maintenance [1]. Increasingly, mobile tunnel scanning systems (MTSS) are being used to monitor structural conditions inside tunnels [2]. MTSS has helped reduce maintenance costs and improve safety by efficiently assessing damage to tunnel concrete linings and analyzing data efficiently [3].

In particular, attention has been drawn to damage detection techniques based on deep learning (DL)-based convolutional neural networks (CNNs) by utilizing image data acquired by MTSS [4]. CNNs have the potential to effectively analyze defects in the concrete lining surface of tunnels through techniques such as image classification, object detection, and semantic segmentation. For example, image classification networks such as AlexNet [5], Visual Geometry Group (VGG) networks [6], Inception networks [7], and ResNet [8] have shown promising results in classifying concrete surface damage. However, some networks may underperform due to the diversity of damage types and variability in training data quality [9,10].

Object detection techniques such as Faster Region-Based Convolutional Neural Network (Faster RCNN) [11], Single Shot Multibox Detector (SSD) [12], and You Only Look Once (YOLO) [13] are suitable for detecting defect locations and may enable more accurate damage identification in tunnel structures when linked with MTSS. Xue and Li [11] proposed and designed a Faster RCNN to detect cracks in images obtained from a moving tunnel inspection (MTI-100), a railroad tunnel scanning device, which showed more improved accuracy than GoogLeNet [14], AlexNet, and VGG networks. Although object detection is effective at locating defects, it is limited in its ability to represent their specific contours or shapes in detail [15]. Semantic segmentation techniques have been introduced to address these limitations. Networks such as Fully Convolutional Network (FCN) [16], DeepLab [17], U-Net [18], DenseNet [19], and SegNet [20] have demonstrated strengths in detecting and visually representing defects at the pixel level. Huang et al. [21] upgraded the existing MTI-100 to MTI-200a to enable crack detection in railroad tunnel linings by training an FCN model. However, in terms of damage detection, the performance in identifying and classifying cracks, exfoliation, and leakage locations was improved, but it remained insufficient for assessing the structural state. Furthermore, issues concerning dataset collection and the quality of images collected at high speeds remain to be addressed for quantitative assessment of damage [22].

Although CNN-based algorithms successfully detect cracks, several technical issues remain [23]. The performance of CNN-based damage detection depends on crack image quality under different conditions [24]. Images collected by MTSS may deteriorate due to motion blur (MB) and poor resolution caused by vehicle vibration and high-speed movement [25]. Differences in operator skill during data collection and processing can influence dataset quality [26], limiting high-precision tasks such as micro-crack detection. Research on data collection and quality is needed to improve DL model performance by quantitatively evaluating MTSS image quality.

Image quality assessment (IQA) comprises subjective and objective methods [27]. The former relies on human vision but is impractical in many image processing applications. The latter quantifies quality through a numerical matrix and is classified into full-reference (FR), reduced-reference (RR), and no-reference (NR) IQA, based on reference image dependence [28]. FR-IQA measures the difference between reference and distorted images [29], and RR-IQA utilizes reference image information such as metadata or distortion type [30]. NR-IQA predicts the degradation level of distorted images without reference image information [31]. As NR-IQA requires no reference image, it is practical and applicable to real-world environments [32]. Because securing undistorted reference images is difficult in real applications, NR-IQA is essential for MTSS.

In this study, we focused on the idea that image quality affects the performance of DL, and that using high-quality images can improve DL performance. Assessing the applicability of NR-IQA in MTSS to classify low-quality images caused by MB during image data collection and training is necessary. However, precisely estimating the point spread function (PSF) to determine the directionality of MB caused by mobile equipment such as MTSS is challenging due to various factors, including camera vibration, vehicle speed, and exposure time [33]. To address these issues, instead of PSF-based approaches, this study proposes an approach using directional Gaussian blur to simulate the horizontal characteristics of MB that may occur in MTSS and to use it as an evaluation tool.

In this study, we examined the applicability of NR-IQA quality assessment metrics for MB image evaluation in MTSS and CNN-based damage detection for tunnel maintenance. We utilized an image dataset acquired by MTSS and the publicly available Kaggle crack dataset [34], generated horizontal MB images, and analyzed the correlation between NR-IQA-based image quality metrics and the crack detection performance of CNN models. Furthermore, we examined the appropriateness of NR-IQA quality metrics, defined a threshold range for classifying low-quality images, and attempted to derive MTSS dataset management measures to improve DL performance.

## 2. Related Research

### 2.1. Limitations of Reference-Based IQA (FR and RR)

In image quality assessment (IQA), mean squared error (MSE) was once the de facto standard for evaluating restoration quality. However, it has been criticized for its weak alignment with human perception—images with the same MSE can be judged very differently by observers [35]. To mitigate this limitation, peak signal-to-noise ratio (PSNR) and structural similarity index (SSIM) [36] are widely employed as full-reference (FR) metrics in deblurring and restoration.

PSNR computes a logarithmic ratio of the maximum signal power to the noise power derived from MSE, and is often used to assess degradation due to additive noise [37]. In practice, compared to SSIM, PSNR tends to respond more strongly to noise reduction and mild Gaussian blur [38]. In contrast, SSIM compares luminance, contrast, and structure components between an original and a distorted image. It is particularly sensitive to structure-preserving distortions and is often more consistent than PSNR with perceived quality loss due to JPEG/JPEG2000 compression artifacts [39]. Because they emphasize different aspects of distortion, PSNR and SSIM are commonly used as complementary metrics.

To date, several SSIM-derived FR-IQA methods have been proposed, including multi-scale SSIM (MS-SSIM) [40] and complex wavelet SSIM (CW-SSIM) [41]. MS-SSIM aggregates similarity across spatial scales and is widely used in display and compression quality assessment; however, it is not specifically optimized for motion blur (MB). CW-SSIM, which relies on phase consistency in a complex wavelet domain and is robust to small translations and rotations; however, this robustness can limit its ability to directly quantify the progressive loss of high spatial frequencies caused by MB. To address this shortcoming, Abdullah-Al-Mamun et al. [42] proposed the blur level (BL) metric that captures blur induced by small pixel shifts and rotations—effects that CW-SSIM tends to ignore. While BL often outperforms SSIM and PSNR in describing motion blur and sharpness, especially in low-light or low-texture scenes, it still does not provide directional information about the blur.

A fundamental limitation of FR-IQA methods is their dependence on a pristine reference image. In practical tunnel inspection with MTSS, high-speed motion renders simultaneous acquisition of a clear reference infeasible, which results in the absence of an absolute target for evaluating blurred images.

As RR-IQA, the modulation transfer function (MTF) is a widely accepted physical measure of sharpness in vision-based camera systems [43]. MTF characterizes the frequency response of imaging systems—including microscopy, radiography, and remote sensing [44,45,46,47]—and is the magnitude of the optical transfer function (OTF), where the OTF is the Fourier transform of the point spread function (PSF) that describes blur. Consequently, spatial resolution can be assessed via the high-frequency behavior of the MTF [48].

Previously, MB effects have been investigated based on MTF changes. Dinh et al. [43] used an indoor rotational rig and observed that increasing exposure broadens the PSF (i.e., shallower edge spread and reduced MTF). Luo et al. [49] recorded video while translating a mobile phone at 1 m/s, likewise observing PSF broadening and MTF reduction with increasing speed.

However, MTF measurement typically requires specialized test charts and standardized procedures, and it does not readily capture directional blur in natural scenes—constraints that limit its practicality for deep learning pipelines built on large-scale natural images or public datasets [42]. These limitations are amplified in high-speed translational settings such as MTSS, where conventional MTF measurements struggle to reflect the true characteristics of operational motion blur. To bridge this gap, a tunnel-simulated indoor testbed with controlled camera motion, exposure, and illumination can be utilized for acquiring motion-blurred data and accurately characterizing PSF-related behavior for subsequent modeling and learning.

### 2.2. Potential of No-Reference Image Quality Assessment (NR-IQA)

Previous studies on NR-IQA have indicated the importance of considering the characteristics of entire areas in balance through the collection and merging of local image data. Accordingly, the NR-IQA technique can be categorized into statistical estimation methods based on Natural Scene Statistics (NSS) and data-driven learning methods utilizing the Mean Opinion Score (MOS), a subjective image quality evaluation score. In particular, NR-IQA models that apply neural networks such as CNNs and Transformers as regressors have been actively researched [50].

Data-driven NR-IQA studies have suggested modeling the human visual system (HVS) to evaluate image quality by considering local and entire areas [51]. Such cognitive modeling-based IQA shows high sensitivity to specific distortion types, but has limitations in quantitatively estimating complex distortion patterns and semantic image deterioration [52]. Thus, previous studies have utilized image data with the subjective quality evaluation score, MOS, and mapping quality scores using CNN.

CNN-based IQA models have shown high performance by effectively capturing hierarchical characteristics and complex local distortion patterns in distorted images [53]. However, while this approach is strong in extracting low- and medium-quality image distortion features, it has limitations in comprehending semantic features or the broader image context, which requires higher quality. Transformer-based IQA models have been recently introduced to compensate for such limitations, but have mainly focused on analyzing structural features in images and have not been fully utilized to derive perceptual features for evaluating image quality [54,55].

The performance of NR-IQA models can mainly be evaluated by utilizing publicly available datasets. Representative datasets include the Laboratory for Image and Video Engineering (LIVE) [56], Categorical Subjective Image Quality [57], Tampere Image Database 2008 (TID2008) [58], Tampere Image Database 2013 (TID2013) [59], Blurred Image Dataset (BID) [60], and Camera Image Database (CID2013) [61]. With these datasets, the performance of NR-IQA metrics is generally evaluated using such measures as Pearson’s linear correlation coefficient (PLCC), Spearman’s rank correlation coefficient (SROCC), Kendall rank correlation coefficient (KROCC), and root mean square error (RMSE). These metrics are used to validate how well the results of image sharpness evaluation algorithms align with human subjective judgment [62].

Cracks have a continuous line structure from the perspective of an entire area, which makes it unnecessary to consider whole-area characteristics by collecting and merging local data (as done in NSS-based datasets in previous NR-IQA studies). However, publicly available concrete crack datasets such as Kaggle, SDNET2018 [63], METU [64], and Historical-crack18-19 [65] have been constructed to evaluate the performance of deep learning models, but quantitative scores for the quality of crack images have not been reported.

Studies utilizing NSS-based datasets and the NR-IQA technique have been conducted to improve CNN-based crack detection in concrete structures. Pennada et al. [66] used the Blind/Referenceless Image Spatial Quality Evaluator (BRISQUE) [67] to classify low-quality images affected by noise and blur, analyzing the correlation between VGG16 model performance and image quality deterioration. They confirmed improved model performance in high-quality datasets compared to those with noise and blur. However, for horizontal motion blur (MB) images occurring along the driving direction in MTSS environments, the consistency between BRISQUE scores and actual image quality may decrease [68]. Pennada et al. [66] proposed removing low-quality images by setting a threshold below 45 in the BRISQUE range of 1 (sharp) to 100 (blurry), and implementing management measures to construct high-quality datasets.

The essential steps of a deep learning system are data collection, data cleaning and validation, robust model training, and deployment. As the importance of data-centric AI has been recently highlighted, more studies have focused on enhancing model accuracy by optimizing data pre-processing rather than improving learning model algorithms [69,70]. Therefore, to secure CNN-based crack detection performance in images obtained by MTSS, developing a database management method is necessary. This method should maintain the consistency of high-quality data by removing low-quality images after utilizing NR-IQA-based quantitative image quality scores during data collection or cleaning and validation, which precedes model training, rather than developing existing algorithms.

## 3. Test Design

### 3.1. MB Image Datasets

In this study, we applied an alternative MB image generation method using a Gaussian blur filter, instead of Point Spread Function (PSF)-based MB modeling, to quantitatively evaluate the motion blur (MB) phenomenon occurring in a tunnel environment with MTSS by utilizing NR-IQA, and to analyze the impact of such blur on the performance of the crack detection model.

In general, an MTSS travels at high speeds inside a tunnel to capture the surface of the concrete lining, and in this process, it likely causes horizontal blur depending on the travel speed of the vehicle, the distance between the camera and the subject (tunnel wall), and the exposure time of the camera. Such MB becomes more intense at higher travel speeds and longer exposure times and, consequently, is highly likely to distort fine features such as cracks and defects on the tunnel surface, degrading the performance of automated crack detection models.

Previous studies have applied blur models based on PSF to simulate these blurs, enabling accurate reflection of MB characteristics [71,72]. However, in real tunnel environments, precisely estimating PSF is difficult due to complex interactions of structural and environmental factors, and lighting conditions, camera sensors, and vehicle vibration make it challenging to maintain consistent PSF patterns [73]. These problems have made the consistent application of PSF-based MB models difficult in practice, limiting their use in tunnel inspection systems.

We suggest simulating MB in MTSS environments by applying a horizontal Gaussian blur filter, instead of directly estimating the PSF. The Gaussian blur filter spreads blur effects to surrounding pixels in images and can control blur intensity quantitatively. Rather than a conventional Isotropic Gaussian Blur, we applied a filter that spreads blur only horizontally to better reflect MB characteristics in MTSS.

We specifically simulated horizontal MB because in prior field experiments by Lee et al. [74], eSFR charts were affixed to tunnel walls to enable MTF analysis and compare horizontal and vertical blur during MTSS operation. The results showed that blur occurred more severely in the horizontal (driving) direction than in the vertical direction because of vehicle vibration.

In this study, we utilized the publicly available Kaggle crack datasets and tunnel datasets acquired by MTSS, generated various levels of MB images, and analyzed the impacts of the image quality of NR-IQA depending on the blur intensity on the performance of crack detection models. In detail, we generated image data by applying a horizontal Gaussian filter while gradually increasing the range of the blur spreading from left to right based on the center pixel, from 10 to 50.

The algorithm (Equation (1)) for assigning the MB effect to an image, and the specific generation method are as follows.

(1) $$k=\frac{1}{3}[\begin{array}{ccc} 0 & 0 & 0\\ 1 & 1 & 1\\ 0 & 0 & 0 \end{array}]$$

1. A k×k forward zero matrix is created as a kernel.
2. 1 is assigned to the center row of the kernel matrix (*k*/2 for even numbers, and *k*/(*k* + 1) for odd numbers).
3. The average is calculated after dividing the entire value of the kernel matrix by *k*.
4. An operation is performed on the input image by a convolution of a *k* × *k* kernel.
5. A value of 1/size is distributed in the center row, and a convolution is performed to average the left and right pixels in the horizontal direction to create a horizontal blur effect.

**Figure 1:**
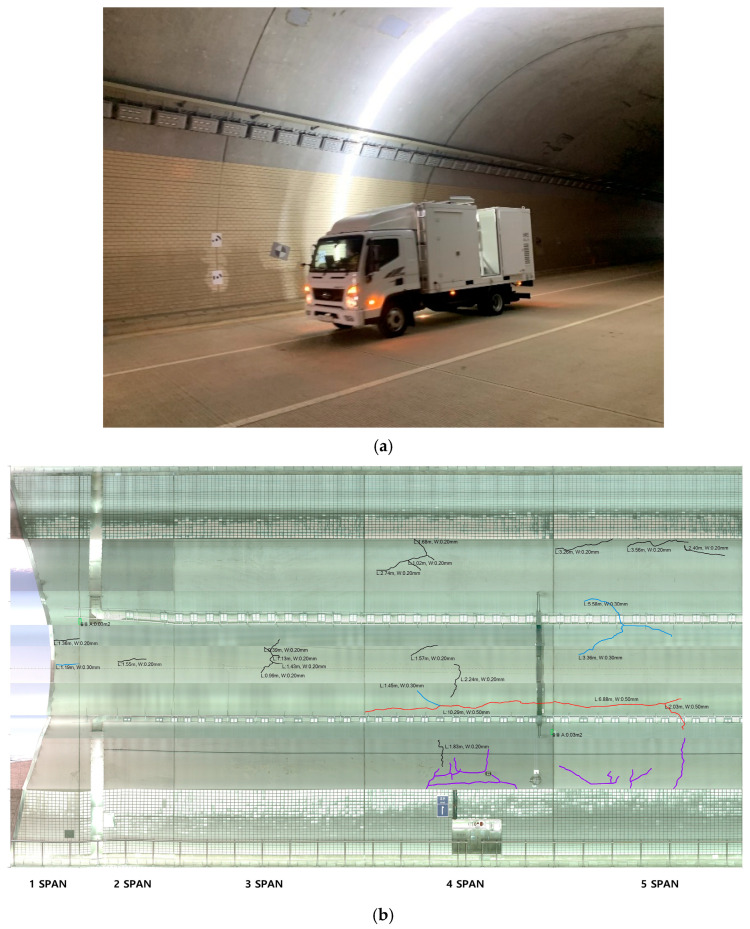
MTSS dataset collection methods. (**a**) Mobile tunnel scanning system (MTSS). (**b**) Images that detected cracks in a tunnel’s concrete lining acquired by MTSS.

The MTSS dataset utilized in this study was constructed by refining image data obtained by MTSS in a real tunnel environment (Figure 1). The MTSS used in the data collection process is equipped with a 4K resolution line-scan camera, which can capture the inside of a tunnel at a maximum speed of 80 km/h with a resolution of 1.0 mm/pixel. The standoff distance between the camera and the tunnel lining is another critical factor governing image quality that requires careful control. In this study, the MTSS was equipped with custom-designed illumination that provided sufficient luminance for line-scan imaging at distances up to 10 m. Because the curved tunnel geometry naturally causes variations in the camera-to-wall distance, the optical configuration was calibrated by replacing the lens to maintain a uniform spatial resolution of 1.0 mm/pixel. This pre-calibration minimized potential degradation in image sharpness and resolution from distance variability. The original images collected in this manner were cleaned and preprocessed, resulting in a dataset of 27,736 images with a size of 512 × 512.

Furthermore, we utilized publicly available crack datasets on Kaggle to evaluate the generalization capability of the CNN model. The Kaggle datasets consisted of 11,298 images with a size of 448 × 448, and the same preprocessing was applied in the test.

In this study, we generated five types of MB images by setting the kernel size k to 10, 20, 30, 40, and 50 for each dataset. In the end, we constructed six datasets (original + five types of MB), including original images. When training the CNN model, we utilized Kaggle datasets by dividing them into 9603 images for training (train images), and 1695 images for testing (test images).

**Figure 2:**
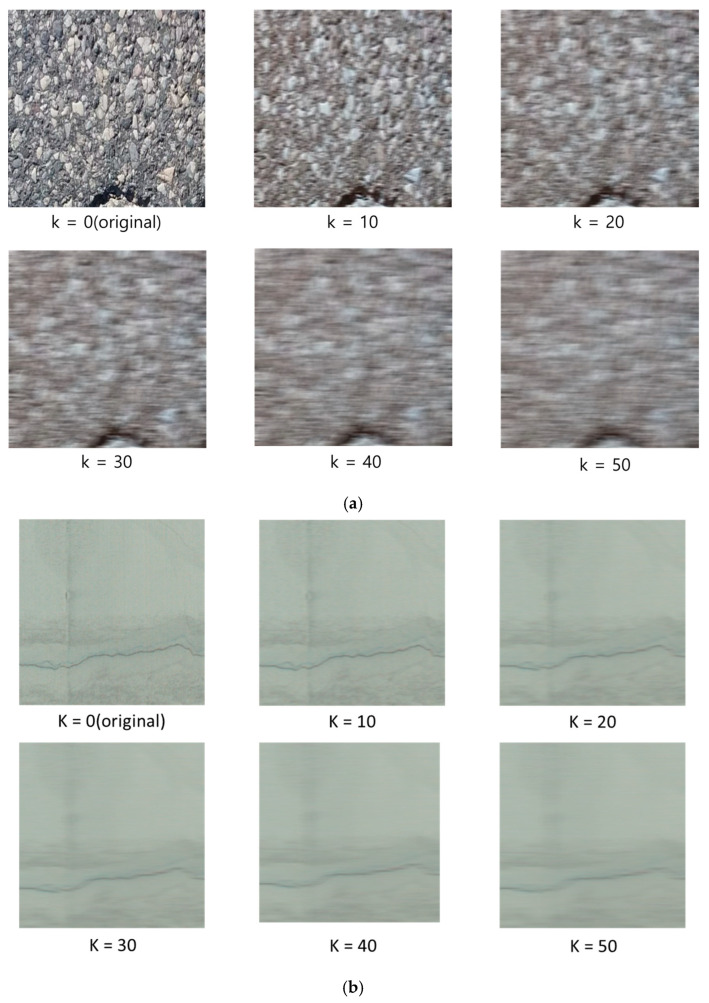
Samples of 12 types of motion blur image datasets. (**a**) Kaggle dataset with five generated motion blur intensities. (**b**)The MTSS dataset generated 5 intensities of motion blur.

In the MTSS dataset, the proportion of images with cracks is extremely low (less than 5% of the total). Thus, random sampling of the test data is highly likely to include an excess of images without cracks. To prevent such a case, and ensure a more balanced distribution of cracks within the test data, we arranged the images in the mask file based on the number of pixels with cracks, and selected 100 images from the top third section with a large number of cracks, 100 images from the middle third section with a moderate number of cracks, and 100 images from the bottom third section with very few cracks. By constructing the test data after randomly sampling from each section, we adjusted the distribution of cracks to be balanced. Finally, as shown in Figure 2, we constructed a total of 12 datasets for each of the Kaggle and MTSS datasets, including the original images and five types of MB images.

In this study, we utilized the generated MB images to quantitatively evaluate how the performance of the crack detection CNN model shifted by the blur intensity, and eventually to explore how to manage MTSS datasets using NR-IQA, which is applicable in practice in MTSS-based tunnel inspection systems.

### 3.2. CNN Training

The generalization capability of CNN reportedly relies on large-scale labeled training data [75]. Thus, when the amount of training data is insufficient, classification accuracy drops, resulting in an overfitting problem. The outstanding image recognition performance of CNN presumably depends on training with large datasets, but the collection of sufficient and effective data is still practically difficult in maintenance [76].

Transfer learning (TL) has emerged as an efficient approach to overcome such limitations, and it is recognized as a powerful tool to reduce the dependence on the amount of training data [77]. Compared to conventional machine learning (ML) methods, TL divides data into a source domain and a target domain, and learns by applying data learned in the source domain to a related target domain [78].

In this study, we constructed a learning model based on the U-Net architecture, a semantic segmentation model, to analyze crack detection performance via a CNN model with TL. We also used four pre-trained networks, such as ResNet18, ResNet34, VGG11, and AlexNet, as the backbone responsible for feature extraction. For modeling training, we applied the Dynamic U-Net, a variant of the U-Net. The Dynamic U-Net is a model that utilizes various encoders to extract the features of input images, and then restores them to a decoder with a symmetrical structure to produce an output with the same size as the original image. In particular, the encoders of U-Net usually utilize pre-trained weights in large datasets such as ImageNet, which can accelerate model training and improve generalization capability [79].

In this study, we utilized Fastai, a deep learning library in Python 3.13, to evaluate the crack detection performance of TL-based CNN models. Fastai is based on PyTorch 2.4 and aids users in easily constructing CNN models with TL [80]. For model training, images from the Kaggle dataset (originally 448 × 448) and MTSS dataset (512 × 512) were uniformly resized to 256 × 256. Image normalization was performed using the ImageNet statistics (mean: [0.485, 0.456, 0.406]; std: [0.229, 0.224, 0.225]) to ensure compatibility with pre-trained backbones.

The dataset was randomly split into 80% training and 20% validation parts, with a fixed random seed for reproducibility. Mask images were binarized with class labels 0 (background) and 1 (crack).

**Figure 3:**
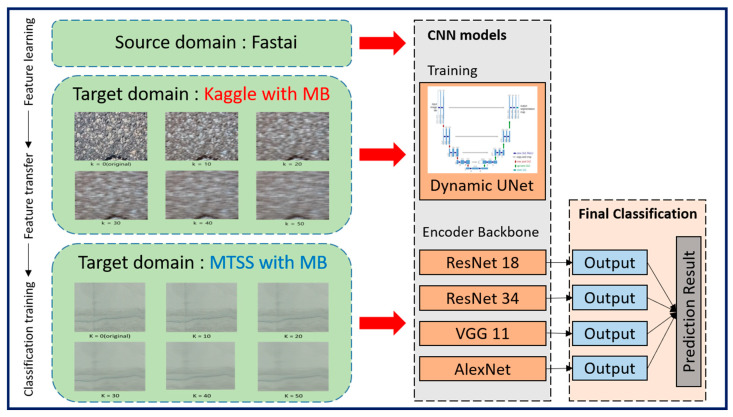
Schematic of crack diagnosis on motion blur images using CNNs.

We employed a dynamic U-Net with ImageNet-1K (Figure 3)—pretrained encoders (ResNet18, ResNet34, VGG11, and AlexNet). The decoder mirrored the encoder and used skip connections to propagate multi-scale feature maps. The final output layer comprised two channels (background, crack) with a softmax activation for pixel-wise classification.

The learning rate was selected via an automated search by increasing the rate logarithmically from 1 × 10^−6^ to 1 × 10^−1^ and identifying the “valley point” with the steepest drop in loss. Training then adopted the one-cycle policy: the LR was raised to its maximum during the first 25% of epochs and decayed over the remaining 75%, with momentum cycled at 0.95 → 0.85 → 0.95. Using these settings, we compared the crack-detection performance across networks and analyzed the correlation between the NR-IQA scores and CNN performance on MB images.

## 4. Test Results and Analysis

### 4.1. CNN Training Results

We employed statistical metrics, including recall, selectivity, precision, accuracy, and F1-score, to analyze the crack detection performance of different CNN models. Recall refers to the percentage of true positive cases correctly predicted by the model. Accuracy is the percentage of correctly predicted cases among all cases, and precision is the percentage of cases that are actually positive among cases predicted as positive. Selectivity is the percentage of measuring the model’s performance in identifying *TN* (true negative), and F1-score is a harmonic mean of recall and precision, i.e., an evaluation metric considering the balance between the two metrics. A higher value of an evaluation metric indicates a better performance of the evaluated model. The mathematical expression for these metrics can be found in the following equations:

(2) $$Recall=\frac{TP}{TP+FN}$$

(3) $$Selectivity=\frac{TN}{TN+FP}$$

(4) $$Precision=\frac{TP}{TP+FP}$$

(5) $$Accuracy=\frac{TP+TN}{TP+TN+FP+FN}$$

(6) $$F1=\frac{2TP}{2TP+FP+FN}$$

where true positive (*TP*) refers to the number of cases where the model correctly predicts as positive, false positive (*FP*) is the number of cases where the model incorrectly predicts as positive, *TN* is the number of cases where the model correctly predicts as negative, and false negative (*FN*) refers to the number of cases where the model incorrectly predicts as negative.

**Figure 4:**
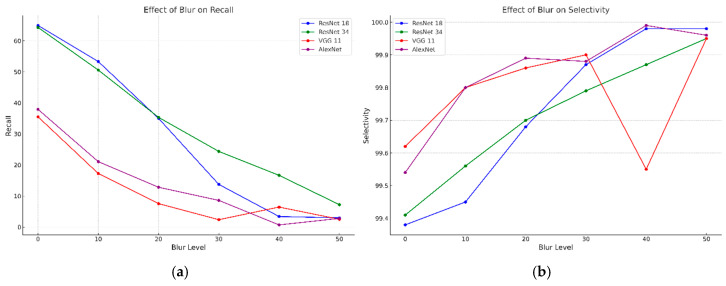

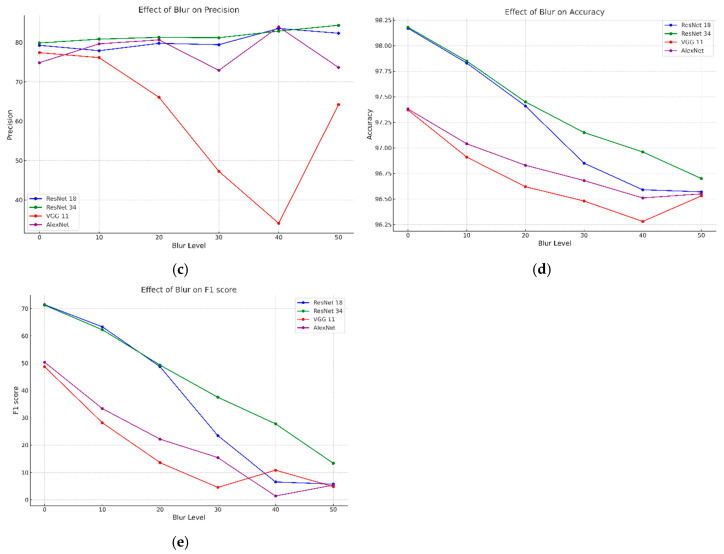
CNN training results for MB intensity of 0–50 on the Kaggle dataset. (**a**) Recall. (**b**) Selectivity. (**c**) Precision. (**d**) Accuracy. (**e**) F1-score.

We analyzed the crack detection performance of the four CNN models based on the confusion matrix, utilizing images generated by assigning MB intensities of 10–50 to the Kaggle dataset. Table 1 and Figure 4 present the evaluated recall, selectivity, precision, accuracy, and F1-score. In Figure 4, the *y*-axis shows the CNN evaluation metrics, and the *x*-axis denotes the MB intensity.

All the models showed a rapid downward tendency of recall as the blur intensity increased, indicating the limitation of detecting cracks with increasing blur intensity (Figure 4a). In particular, the recall of ResNet18 was 64.96% at blur 0, but decreased to 2.97% at blur 50, and that of ResNet 34 also decreased from 64.32% to 7.23%. The recall of VGG11 and AlexNet decreased even more radically, with a drop to 2.79% at blur 50 in terms of AlexNet. These findings imply the dramatic drop of the CNN model’s capability of detecting cracks in highly blurred images, with VGG11 and AlexNet being particularly less tolerant to blurring.

All the models maintained a high selectivity of over 99% even as the blur intensity increased (Figure 4b). This result indicates that the model’s capability to accurately exclude crack-free areas is not affected. However, the rapid decrease in recall indicates that the model is more inclined to correctly exclude non-cracks, instead of being unable to detect cracks.

Precision remained relatively constant with increased blur intensity, showing differences between models. As shown in Figure 4c, ResNet18 and ResNet34 precision slightly increased with blur intensity, likely due to fewer false positives. The precision of VGG11 was 77.41% in the original images but decreased to 64.21% at blur 50, showing a significant reduction compared to other models. AlexNet maintained stable precision but became volatile after blur 40. These results suggest that as blur intensity increases, fewer detectable cracks maintain precision levels.

In Figure 4d, accuracy shows a downward tendency as blur intensity increases; ResNet18 and ResNet34 accuracy decrease from 98.17% and 98.18% to 96.57% and 96.70%, respectively, and VGG 11 and AlexNet show similar patterns, maintaining a lowest accuracy of 96.51%. However, given the high selectivity and limitation in evaluating crack detection performance based on accuracy alone, analysis of recall and F1-score is necessary.

As illustrated in Figure 4e, the F1-score drastically decreases as the blur intensity increases, with the F1-score of ResNet18 dropping from 71.41% to 5.73%, and that of ResNet34 decreasing from 71.25% to 13.31%. F1-score of VGG11 and AlexNet recorded extremely low values of 4.82% and 5.37%, respectively, at blur 50, which implies that the CNN models are practically ineffective at detecting cracks in highly blurred environments. The large decrease in the F1-score of VGG11 and AlexNet especially indicates that these models are more vulnerable in highly blurred images.

**Figure 5:**
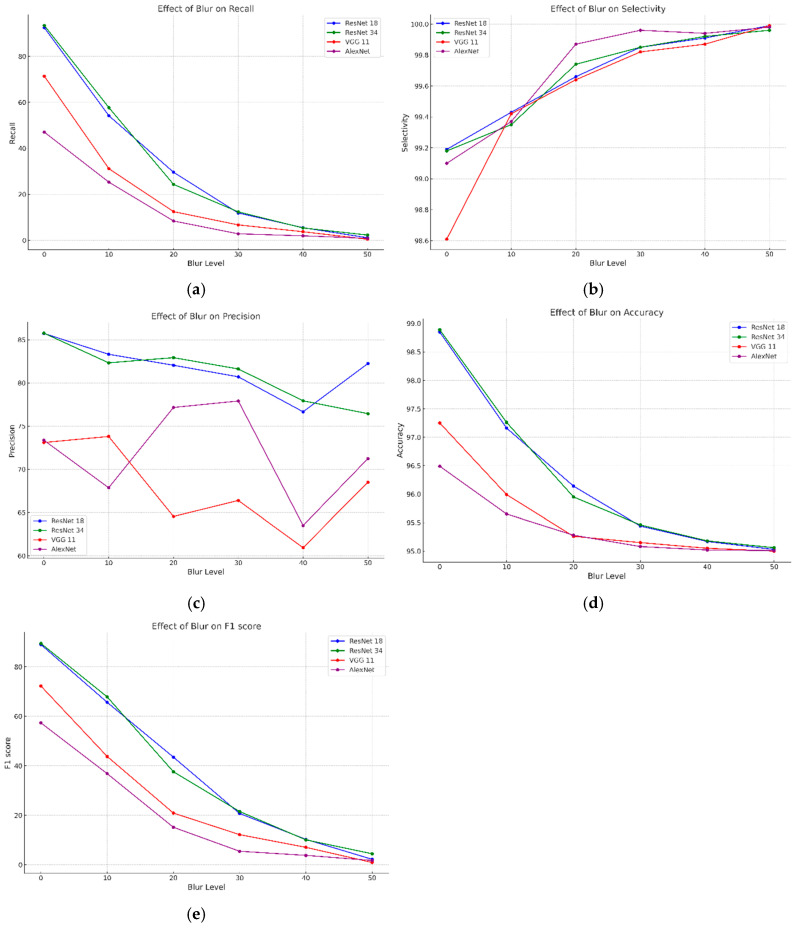
CNN training outcomes for MB intensities of 0–50 on the MTSS dataset. (**a**) Recall. (**b**) Selectivity. (**c**) Precision. (**d**) Accuracy. (**e**) F1 score.

Table 2 and Figure 5 illustrate the crack detection performance results for the four CNN models by utilizing images generated from the MTSS datasets with an MB intensity of 10–50: recall, selectivity, precision, accuracy, and F1-score.

As presented in Figure 5a, as the blur intensity increased, the recall of all models drastically decreased, indicating that the more intense the blur, the less frequently cracks are detected. In particular, the recall of ResNet18 decreased from 92.46% to 1.12%, that of ResNet34 dropped from 93.41% to 2.29%, and those of VGG 11 and AlexNet showed an even faster downward tendency, plunging from 71.36% to 0.47% and from 47.05% to 0.88%, respectively. These results suggest that CNN models are ineffective at extracting crack features from blurred images.

All the models maintained a high selectivity of higher than 99% regardless of the blur intensity (Figure 5b), implying that an increase in blur does not significantly affect the capability of identifying crack-free areas. However, when considered in conjunction with the rapid decrease in recall, the model tended to accurately exclude non-cracks instead of being unable to detect cracks.

As illustrated in Figure 5c, precision did not show a rapid change even with increasing blur intensity, with most models maintaining relatively constant values. For instance, the precision of ResNet18 slightly decreased from 85.74% to 82.25%, that of ResNet34 also dropped from 85.77% to 76.44%, and those of VGG11 and AlexNet also showed a relatively small decrease. These outcomes indicate that the models can provide reliable results when detecting cracks, but cannot detect them when the blur increases.

All the models showed decreased accuracy with increasing blur intensity (Figure 5d). The accuracy of ResNet18 decreased from 98.85% to 95.03%, that of ResNet3 decreased from 98.89% to 95.06%, and those of VGG11 and AlexNet decreased faster, dropping from 97.25% to 95.00% and 96.49% to 95.01%, respectively. The ResNet models maintained higher accuracy for blurring, while VGG11 and AlexNet degraded more quickly.

As shown in Figure 5e, the F1-score is the metric that decreases most rapidly with increasing blur intensity. F1-score of ResNet18 plunged from 88.97% to 2.21%, and that of ResNet34 from 89.43% to 4.45%. The F1-score of VGG11 and AlexNet showed even more severe declines, plunging from 72.22% to 0.93% and from 57.33% to 1.74%, respectively. These outcomes show that the models lost their capability of detecting cracks as the blur increased.

**Figure 6:**
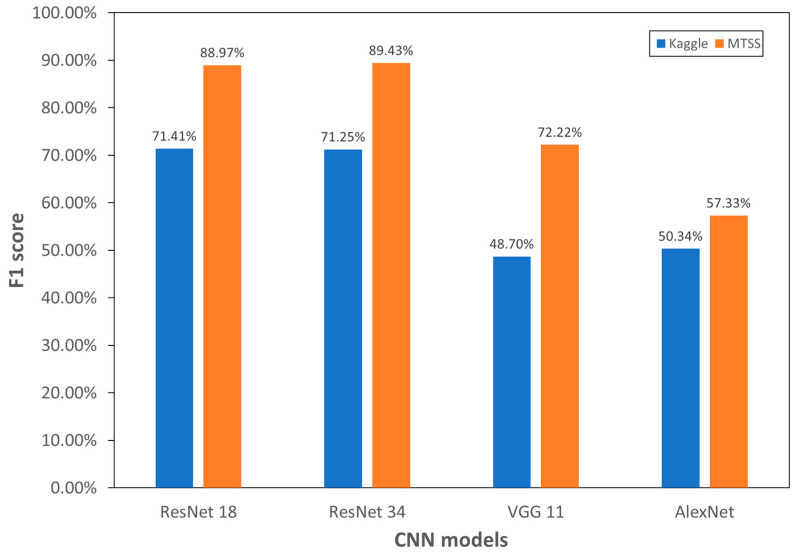
Comparison of crack diagnosis by four CNN models between the Kaggle and MTSS datasets.

Figure 6 compares crack detection performance between the Kaggle and MTSS datasets. Comparing F1-scores on original images shows significant performance differences across datasets, despite using the same training model. The F1-score of ResNet18 was 71.41% on Kaggle and 88.97% on MTSS, showing a 17% difference. ResNet34 showed an 18% performance difference, VGG11 around 23%, and AlexNet about 7%. On the Kaggle dataset, the F1-score (71.41%) of ResNet18 exceeded that of ResNet34 (71.25%), whereas in MTSS, ResNet34 (89.43%) outperformed ResNet18 (88.97%). VGG11 (72.22%) in MTSS showed higher values than those of ResNet18 (71.41%) and ResNet34 (71.25%) in Kaggle. VGG11 and AlexNet, showing similar performance on Kaggle, exhibited different trends on MTSS. VGG11 improved by over 24%, while AlexNet improved by 7%. These results indicate that CNN-based crack detection performance varies significantly based on image data quality. Thus, dataset quality is crucial for the CNN model’s crack detection performance.

The findings of this study showed that the performance of CNN-based crack detection models degraded drastically as the Gaussian blur intensity increased on the Kaggle and MTSS datasets. ResNet18 and ResNet34 were relatively more resistant to increasing blur, but their performance eventually dropped at high levels of blur intensity. VGG 11 and AlexNet were vulnerable to increased blur, and especially, the huge decreases in the recall and F1-score indicate significant degradation of actual crack detection performance. While precision is relatively constant, considering more cases where detection itself is impossible with increasing blur is crucial. Accuracy did not decrease significantly because of high selectivity, reflecting the lower performance of actual crack detection. In particular, the model’s capability of detecting cracks became almost neutralized in the case of severe blurring, as F1-score values plunged.

### 4.2. Correlation Between NR-IQA and CNN-Based Crack Detection Performance

We found that the crack detection performance of the CNN models degraded due to MB in the images. We utilized NR-IQA metrics such as BRISQUE, Naturalness Image Quality Evaluator (NIQE) [81], Perception-based Image Quality Evaluator (PIQE) [82], and Cumulative Probability of Blur Detection (CPBD) [83], to evaluate the quality of these blurred images. BRISQUE, NIQE, and PIQE are measured on a scale from 0 (sharp) to 100 (blurry), and CPBD is measured on a scale from 0 (blurry) to 1.0 (sharp). We selected these metrics because they have defined categories for blurriness and sharpness of images, and make it easy to set threshold ranges for image quality scores through correlation analysis with deep learning performance.

Table 3 and Table 4 present the evaluation results of ResNet34 on the Kaggle dataset and the MTSS dataset. We utilized these outcomes and analyzed the correlation between the F1-score of the CNN model and the NR-IQA image quality metrics. The NR-IQA metric values shown in Table 3 and Table 4 represent the average of the image quality metrics measured on the test data in each dataset, and these are utilized in the quantitative evaluation of the impacts of image quality on the crack detection performance of CNN models (for reference, histograms of PIQE and CPBD versus MB intensity for the Kaggle and MTSS datasets are provided in Appendix B).

**Figure 7:**
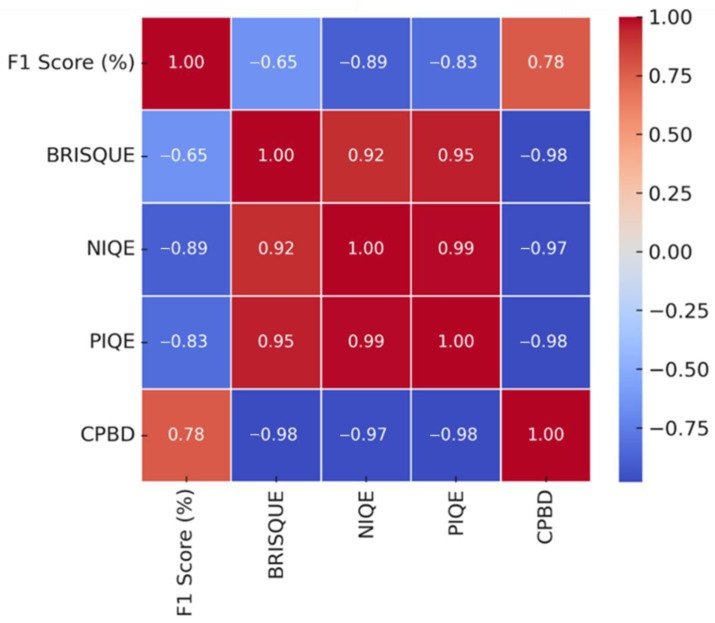
Correlation between the NR-IQA metrics and crack detection performance (F1-score) on the Kaggle dataset.

Figure 7 shows Pearson correlation coefficients between F1-score and NR-IQA metrics for analyzing image quality impact on CNN-based crack detection performance on the Kaggle dataset. The F1-score and NR-IQA metrics, NIQE (−0.89) and PIQE (−0.83), have a high negative correlation, indicating that lower image quality results in reduced crack detection performance. F1-score and BRISQUE show a negative correlation of −0.65. CPBD (0.78) correlates positively with F1-score, indicating that less blurred images led to improved CNN performance. The NR-IQA metrics NIQE (−0.89), PIQE (−0.83), CPBD (0.78), and BRISQUE (−0.65) show high correlation with F1-score.

**Figure 8:**
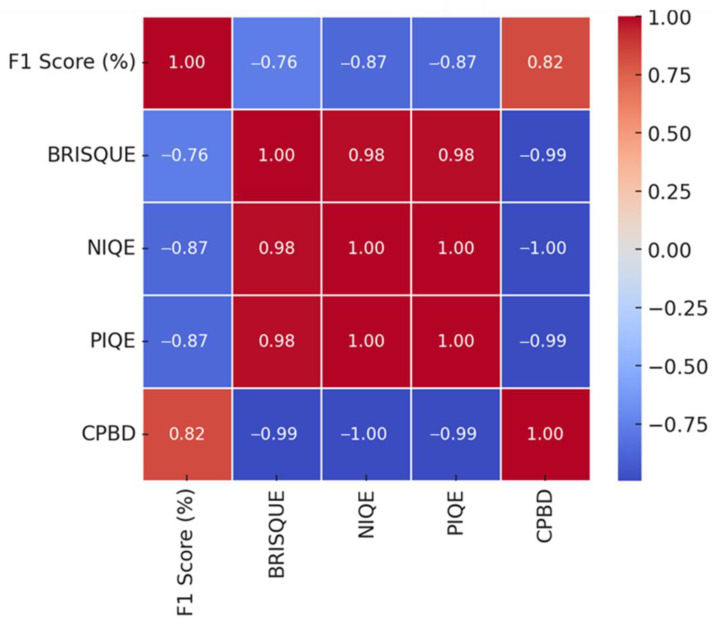
Correlations between NR-IQA metrics and crack detection performance (F1-score) on the MTSS dataset.

Figure 8 illustrates the Pearson correlation coefficients between the F1-score and various NR-IQA metrics on the MTSS dataset for analyzing the impact of image quality on CNN-based crack detection performance. As shown in Figure 8, NIQE (−0.87), PIQE (−0.87), and BRISQUE (−0.76) have strong negative correlations with F1-score, showing similar patterns in the Kaggle dataset. Furthermore, CPBD (0.82) has a strong positive correlation with the F1-score, with a larger impact than on the Kaggle dataset. The NR-IQA evaluation metrics, i.e., NIQE (−0.87), PIQE (−0.87), CPBD (−0.82), and BRISQUE (−0.76), in this order, have a high correlation with the F1-score.

These results suggest the possibility of maximizing CNN performance by ensuring optimal image quality in MTSS through NR-IQA metrics. Therefore, utilizing NR-IQA metrics with high correlation with the F1-score is useful for maximizing CNN-based crack detection performance, and optimal image quality evaluation and selection strategies can be set based on the most influential metrics in each dataset. Therefore, setting quality threshold ranges using NR-IQA metrics, as well as a strategy of deleting images that do not meet the criteria, is necessary. Such a method is expected to reduce detection errors caused by highly blurred images and contribute to deriving highly reliable crack detection results.

### 4.3. Linear Regression Analysis of NR-IQA Evaluation Metrics

In the Kaggle dataset, the metrics that are highly correlated with the F1-score are NIQE (−0.88), PIQE (−0.83), CPBD (0.78), and BRISQUE (−0.65), in this order. In the MTSS dataset, the metrics are NIQE (−0.87), PIQE (−0.87), CPBD (0.82), and BRISQUE (−0.76), in this order. Additionally, to select the most appropriate NR-IQA metrics for the two datasets and set a threshold range with the corresponding metrics, based on the F1-score and the regression results for each metric, the threshold range can be set considering the metrics with the highest intensity of change in quality score for each metric for MB.

**Figure 9:**
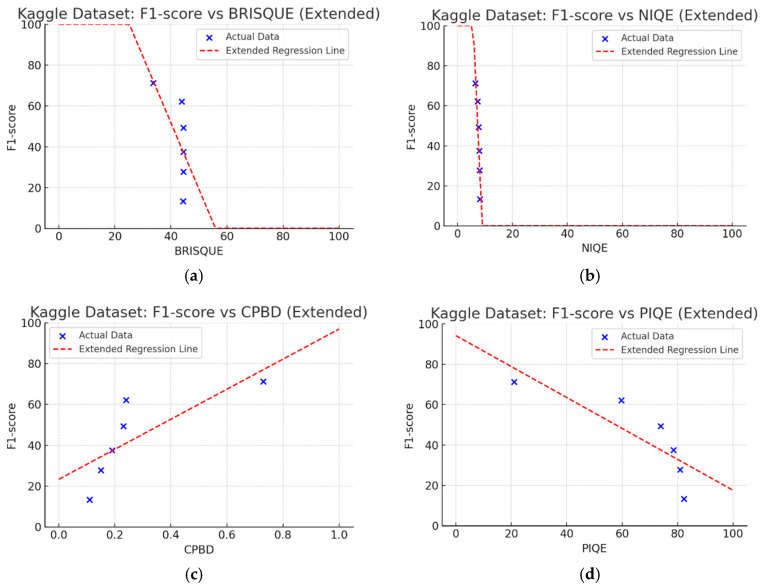
Graphs of linear regression results between F1-score and NR-IQA on the Kaggle dataset. (**a**) Coefficient: 3.25, *p*: 0.1597, R2: 0.42. (**b**) Coefficient: 31.26, *p*: 0.017, R2: 0.79. (**c**) Coefficient: 0.74, *p*: 0.0702, R2: 0.60. (**d**) Coefficient: 0.77, *p*: 0.0397, R2: 0.69.

We performed linear regression to analyze the CNN’s F1-score change with MB intensity for each quality metric in the Kaggle dataset (Figure 9). The *X*-axis had the range of unique scores for each quality metric. Analyzing regression coefficients, CPBD was 0.74, PIQE was 0.77, BRISQUE was 3.25, and NIQE was 31.26. For the coefficient of determination (R2), NIQE had the highest value of 0.79, followed by PIQE (0.69), CPBD (0.60), and BRISQUE (0.42). For *p*-value, NIQE was 0.017, and PIQE was 0.0397, which are lower than 0.05, indicating statistical significance.

**Figure 10:**
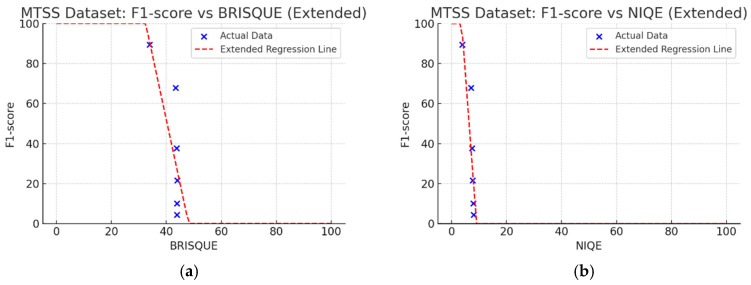

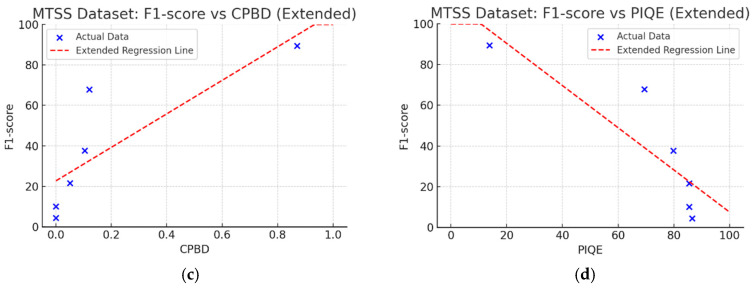
Graphs of linear regression results between F1-score and NR-IQA on the MTSS dataset. (**a**) Coefficient: 3.69, *p*: 0.0774, R2: 0.58. (**b**) Coefficient: 18.45, *p*: 0.0245, R2: 0.76. (**c**) Coefficient: 0.83, *p*: 0.0434, R2: 0.68. (**d**) Coefficient: 1.03, *p*: 0.0247, R2: 0.75.

Figure 10 shows linear regression results on the MTSS dataset. Regression coefficients showed CPBD was 0.83, PIQE was 1.03, BRISQUE was 3.69, and NIQE was 18.45. For the coefficient of determination (R2), NIQE had the highest value of 0.76, followed by PIQE (0.75), CPBD (0.68), and BRISQUE (0.58). The *p*-values were NIQE (0.0245), PIQE (0.0247), and CPBD (0.0434), all below 0.05, indicating statistical significance.

We aimed to devise and develop a dataset management plan that could maintain the consistency of high-quality data by removing low-quality images below a certain level after utilizing NR-IQA-based quantitative image quality scores during data collection, cleaning, and verification in MTSS. The correlation analysis revealed that NIQE had the highest correlation in both the Kaggle and MTSS datasets. However, in the regression analysis, the regression coefficient was 31.26 for the Kaggle dataset and 18.45 for the MTSS dataset, indicating that the F1-score changed significantly when the NIQE score changed by 1. Although image scores of NIQE range from 0 (sharp) to 100 (blurry), the variation in quality scores in the original images is rated around 1, which does not indicate a significant change in image quality. However, in the Kaggle dataset, NIQE was measured to be 6.42 in the original images and 8.09 in the MB 50 images; in the MTSS dataset, it was measured to be 3.82 in the original images and 8.06 in the MB 50 images. Therefore, the NIQE metric cannot be regarded as an appropriate evaluation metric because it cannot fully reflect the variation in quality scores, consistent with blur intensity.

BRISQUE has an image score range from 0 (sharp) to 100 (blurry); however, in these two datasets, the measured results showed that the quality score varied slightly within 45 points, consistent with changes in the intensity of the MB images. As analyzed by Giniatullina et al. [70], regarding horizontal blur, the consistency between BRISQUE scores and actual image quality was likely to be reduced.

CPBD has an image quality range from 0 (blurry) to 1.0 (sharp). As shown in Table 3 and Table 4, when MB increased by 10, image quality decreased from 0.73 to 0.24 in the Kaggle dataset. In the MTSS dataset, when MB increased by 10, quality scores plunged from 0.87 to 0.12. The F1-score decreased from 72.25% to 62.23% in the Kaggle dataset, and dropped from 89.43% to 67.83% in the MTSS dataset, showing different percentage decreases due to MB impact. This impact is analyzed as statistically insignificant with a *p*-value of 0.0702 in the Kaggle dataset. However, with high sensitivity to MB changes, such as CPBD, setting the MB threshold value may be advantageous as it indicates a high classification effect for sharp images.

PIQE has an image quality score range from 0 (sharp) to 100 (blurry). In the Kaggle dataset, the score for blurring was measured, which increased from 21.04 in the original images, 59.7 in the MB 10 images, to 73.87 in the MB 20 images. In the MTSS dataset, the score soared from 13.77 in the original images to 69.35 in the MB 10 images, and the decrease in the score dropped less with the subsequent increase in MB intensity, similar to CPBD. PIQE also showed the highest results, except NIQE, in correlation and regression analysis. It was highly sensitive to changes in MB, and showed a high consistency even with changes in F1-score.

CPBD and PIQE can be considered appropriate NR-IQA metrics for horizontal MB images, and MTSS image quality can be classified using these metrics.

## 5. Threshold Values for NR-IQA Evaluation Metrics

### 5.1. Histogram Analysis of NR-IQA Metrics for Kaggle and MTSS Datasets

Based on the correlation and linear regression analysis of CNN-based crack detection performance, consistent with higher MB intensities for Kaggle and MTSS datasets, CPBD and PIQE metrics of NR IQA were analyzed as metrics with high sensitivity to blur and high association with F1-score. We utilized these metrics to derive a threshold range of image quality scores applicable to MTSS. The prior correlation and regression analysis is related to the comparison of the average image quality scores of the datasets, and we did not consider the distribution of quality scores across the entire datasets. Therefore, it is necessary to consider a histogram of the scores of each NR-IQA evaluation metric for the datasets.

Table 5 shows histograms of the quality metric scores of CPBD and PIQE for the original images in the Kaggle dataset. The CPBD histogram exhibits that the data shows a non-normal and left-skewed distribution pattern, with a maximum value of 1.0, a minimum value of 0.1047, and a standard deviation of 0.1289. The PIQE histogram shows that the data presents a non-normal and right-skewed distribution pattern, with a maximum value of 86.9515, a minimum value of 2.9123, a mean of 21.0473, and a standard deviation of 15.4365.

Table 6 presents the histograms of the quality metric scores of CPBD and PIQE for the original images in the MTSS dataset. In the CPBD histogram, the data shows a non-normal and left-skewed distribution pattern, with a maximum value of 1.0, a minimum value of 0.511, and a standard deviation of 0.1009. In the PIQE histogram, the data exhibited a non-normal and right-skewed distribution pattern, with a maximum value of 43.1943, a minimum value of 4.4171, a mean of 13.7734, and a standard deviation of 7.6322.

### 5.2. Definition of NR-IQA-Based Threshold in the Kaggle and MITSS Datasets

Although normality is not necessarily required for models such as CNN, the normality of the data can affect the model’s performance to some extent [84]. In particular, when measured with the quality scores of CPBD and PIQE, which have a high sensitivity to MB, CNN performance can be expected to improve when trained on images with a certain level of sharpness. Therefore, preparing criteria of image quality and score range that can be used as a reference to derive thresholds for defining a certain level of quality is crucial.

Venkatanath et al. [82] used the LIVE IQA Database Release 2 [56] to propose the PIQE-based quality scale summarized in Table 7 [85]. Because this scale targets general image quality across diverse distortions, we have used it as a reference point while experimentally identifying and adjusting optimal thresholds tailored to the task of crack detection.

Unlike PIQE, CPBD does not have a literature-defined quality scale. Accordingly, Table 8 presents a hypothesis-driven CPBD scale defined in this study, grounded in the strong linear resemblance to PIQE observed in our prior analyses (Figure 9 and Figure 10) and in the empirical distribution of our datasets (e.g., the means in Table 5 and Table 6). This assumption enables a consistent comparative analysis with PIQE.

In the Kaggle and MTSS datasets, we set the threshold ranges of PIQE as 0–20 (excellent), 21–35 (Good), and 0–35 (Excellent+Good), as well as the threshold ranges of CPBD as 1–0.8 (excellent), 0.81–0.65 (Good), and 1–0.65 (Excellent+Good). Then, as shown in Table 9 and Table 10, we classify the data to identify the change in crack detection performance of the CNN model under the three conditions of threshold ranges. Table 9 indicates the histograms of the threshold ranges of PIQE and CPBD in the Kaggle dataset, while eliminating images that belong outside the range. Table 10 shows the histograms of the threshold ranges of PIQE and CPBD in the MTSS dataset, while eliminating images that belong outside the ranges.

### 5.3. Crack Detection Performance Analysis of CNN Models for NR-IQA Thresholds

We performed deep learning analysis on the test data with categorized threshold ranges for CPBD and PIQE. Table 11 summarizes the F1-score for ResNet18, ResNet34, VGG11, and AlexNet regarding the threshold ranges in the Kaggle dataset. The baseline value of F1-score is the original data (All), and we analyzed the performance change compared to other classification conditions (e.g., Excellent, Good, and Excellent+Good).

When filtering image quality based on CPBD, most CNN models showed decreased F1-scores compared to baseline (All). In the Good-quality dataset, ResNet18 and ResNet34 showed F1-score decreases of −8.19% and −8.15%, indicating reduced performance. In the Excellent quality dataset, the decrease was smaller, with some improvements on VGG11 and AlexNet.

PIQE-based filtering showed the largest improvement on the Excellent quality dataset, with a 4.93% increase in F1-score for ResNet18. However, in the Good quality dataset, several CNN models degraded in performance, with AlexNet showing a −15.11% decrease. These results suggest that image quality filtering using PIQE can improve CNN model performance under certain conditions, but does not consistently benefit low-quality classifications.

Table 12 summarizes the F1-scores for ResNet18, ResNet34, VGG11, and AlexNet regarding a range of thresholds in the MTSS dataset. The baseline data is the original unfiltered data (All), and we evaluated the performance change compared to the filtered dataset.

Evaluation of image quality based on CPBD showed that the performances of ResNet18 and ResNet34 improved by 0.22% and 0.20%, respectively, in the Excellent quality scale, but decreased by −2.51% and −2.75%, respectively, in the Good quality scale. The performances of VGG11 and AlexNet increased by 1.42% and 2.31%, respectively, in the Excellent quality scale, showing a positive impact. However, in the Good quality scale, the performances of VGG11 and AlexNet decreased by −11.99% and −23.77%, respectively, indicating a significant decline. These findings show that although removing low-quality data using CPBD can improve performance, excessive filtering can lead to information loss and reduce the crack detection performance of CNN models.

In the case of PIQE-based filtering, in the Excellent quality scale, ResNet18, ResNet34, VGG11, and AlexNet all maintained a relatively stable F1-score, and several models showed a slight increase in performance. In the Good quality scale, all CNN models showed a downward tendency in F1-score, with AlexNet particularly showing a performance degradation of −23.77%.

In the MTSS dataset, the impact of NR-IQA-based filtering varied across CNN models. CPBD and PIQE filtering in the Excellent quality scale tended to increase the F1-score for some CNN models, but in the Good quality scale, performance decreased. In particular, CPBD-based filtering in the Good quality scale significantly decreased the performance of AlexNet and VGG11, confirming that performance varied depending on the threshold setting.

The impact of NR-IQA-based image quality filtering on CNN models’ crack detection varies by dataset characteristics, CNN structures, and quality thresholds. In CPBD-based filtering, lower performance occurred for both datasets in the Good quality scale, with a major decrease for the Kaggle dataset. The MTSS dataset showed moderate performance degradation for some models, but larger decreases for VGG11 and AlexNet. For PIQE-based filtering in the Excellent quality scale, the Kaggle dataset showed greater improvement than MTSS. In the Good quality scale, CNN models’ performance decreased for both datasets, with AlexNet showing the largest degradation on MTSS.

The findings imply that dataset characteristics should be considered when applying NR-IQA-based filtering, and that CNN model performance is highly likely to degrade when trained on medium-quality data, such as the data in the Good quality scale.

## 6. Discussion

This study is focused on a quantitative analysis of how degradation in MTSS images—particularly horizontal MB arising along the driving direction—affects CNN-based crack detection, and proposed optimization strategies using NR-IQA.

As the MB intensity increases, all the CNN models exhibit degraded crack-detection performance, which is primarily evidenced by the F1 score and recall. For the best-performing model on the MTSS dataset (ResNet34), increasing MB from 0 to 50 reduces the F1 score from 89.43% to 4.45% (−84.98 pp) and recall from 93.41% to 2.29% (−91.12 pp). In contrast, selectivity remains above 99% irrespective of blur, and accuracy decreases only modestly (from 98.89% to 95.06%). This indicates that despite the sharp drop in recall, the model still correctly rejects non-crack regions at a high rate; thus, accuracy alone is inadequate to evaluate effective crack detection under blur.

Several visual constraints must be acknowledged in tunnel inspection. First, illumination is critical: its brightness and uniformity decisively influence the reliability of NR-IQA–based quality assessment. Without consistent lighting, the derived quality scores may not accurately represent the true condition of the tunnel lining. Second, the presence of salient features, such as cracks or irregularities, is equally important. When an image consists predominantly of homogeneous, undamaged regions, the lack of identifiable structures inherently limits NR-IQA metrics, reducing their discriminative capability.

Despite using identical architectures, the MTSS dataset consistently yields higher F1 scores than does Kaggle (e.g., ResNet34: 89.43% vs. 71.25%; VGG11: 72.22% vs. 48.70%). This is attributable to the superior initial image quality in MTSS (lower mean PIQE, higher mean CPBD), underscoring the importance of data-centric AI.

Pearson correlations on the MTSS dataset show strong negative associations between F1 and NIQE/PIQE (−0.87 each) and a strong positive association with CPBD (+0.82). BRISQUE is lower (−0.76) and shows negligible variation (≈43) across blur levels (10–50), suggesting limited sensitivity to horizontal MB. NIQE varies by only 4.24 points (3.82 → 8.06), limiting threshold discrimination. Linear regressions confirm that PIQE (R^2^ = 0.75, *p* = 0.0247) and CPBD (R^2^ = 0.68, *p* = 0.0434) provide statistically significant explanatory power for F1 changes and are thus suitable for horizontal MB assessment.

Applying quality-based filtering using the PIQE/CPBD scales improves performance when retaining only “Excellent” images (PIQE ≤ 20 or CPBD ≥ 0.8): on MTSS, AlexNet increases from 63.24% to 64.70% (+1.46 pp) and VGG11 from 76.25% to 77.33% (+1.08 pp). Conversely, filtering to “Good” quality (e.g., CPBD 0.81–0.65) degrades the performance substantially (e.g., AlexNet − 15.03 pp), implying that medium-quality images can confuse training or that over-filtering removes useful variability.

To minimize confounders from architectural complexity, we focus on well-studied, structurally distinct CNNs (ResNet18/34, VGG11, AlexNet). We exclude more recent families such as EfficientNet [86] and ViT [87] given the risk of overfitting and computational overhead relative to our dataset size.

Nevertheless, several limitations and practical considerations remain. First, MB is synthesized with a Gaussian filter; real MTSS blur may involve compound effects (vehicle vibration, illumination changes). Second, the CPBD scale is hypothesized from PIQE-like behavior and requires further validation. Third, while U-Net’s pixel-wise masks aid interpretability, applying advanced XAI (e.g., Grad-CAM) is expected to further strengthen trust [88]. Fourth, real-time deployment depends on throughput; although NR-IQA is lightweight, CNN inference hinges on GPU capacity; achieving near real-time analysis under high acquisition rates will require lightweight models and capable mobile GPUs. Finally, to test generalizability, EfficientNet-B0/B1 and a fine-tuned ViT-S/16 will be evaluated under the same protocol on blurred images in the future.

Although this study did not validate the framework in a robotic system for one-pass inspection [89], the proposed NR-IQA–based approach is computationally efficient and can be readily integrated into robotic tunnel inspection platforms. Its lightweight nature enables real-time filtering of low-quality images during data acquisition, ensuring that only diagnostically reliable data are passed to the crack detection models. Furthermore, the authors are developing a new tunnel scanning system that will incorporate NR-IQA metrics tailored to its specific hardware, further optimizing its applicability.

## 7. Conclusions

This study addresses the degradation of CNN-based crack detection caused by image MB during high-speed MTSS operation by proposing a data-quality management approach using NR-IQA. The principal scholarly contributions are as follows:

1. We quantitatively demonstrated the impact of horizontally oriented MB during high-speed MTSS operation on the diagnostic accuracy of CNN-based crack detection. The sharp drop in the ResNet34’s F1 score from 89.43% to 4.45% with increasing MB empirically confirms the decisive role of image quality in model capability.
2. By comparatively evaluating NR-IQA metrics, we identified PIQE and CPBD as the most reliable indicators for assessing horizontal MB in the MTSS environment, given their strong correlations with F1 (−0.87 and +0.82; *p* < 0.05). These metrics can serve as objective standards for MTSS data quality management.
3. We validated a proactive data-filtering strategy based on the identified NR-IQA metrics. When the dataset was curated to the “Excellent” tier (PIQE ≤ 20 or CPBD ≥ 0.8), AlexNet’s F1 score increased by 1.46%, demonstrating that data-centric quality criteria at collection/preprocessing can enhance final diagnostic reliability.

In conclusion, this study breaks through the prevailing paradigm that focuses on improving model architectures and emphasizes that a data-centric approach that pre-emptively assures input data quality is indispensable for securing the stability and reliability of MTSS-based automated inspection systems. The proposed NR-IQA–based quality-management framework is expected to provide both academic and practical foundations for further advancement of intelligent infrastructure maintenance technologies.

## Figures and Tables

**Table 1:** Evaluation indices of different backbone models for crack diagnosis using the Kaggle dataset with MB *.

Model—Blur	Evaluation Index				
	Recall	Selectivity	Precision	Accuracy	F1-Score
ResNet 18—0	64.96%	99.38%	79.28%	98.17%	71.41%
ResNet 18—10	53.33%	99.45%	77.90%	97.83%	63.31%
ResNet 18—20	35.03%	99.68%	79.77%	97.41%	48.68%
ResNet 18—30	13.75%	99.87%	79.43%	96.85%	23.44%
ResNet 18—40	3.40%	99.98%	83.55%	96.59%	6.53%
ResNet 18—50	2.97%	99.98%	82.34%	96.57%	5.73%
ResNet 34—0	64.32%	99.41%	79.86%	98.18%	71.25%
ResNet 34—10	50.59%	99.56%	80.84%	97.85%	62.23%
ResNet 34—20	35.35%	99.70%	81.30%	97.45%	49.27%
ResNet 34—30	24.38%	99.79%	81.20%	97.15%	37.51%
ResNet 34—40	16.67%	99.87%	82.88%	96.96%	27.75%
ResNet 34—50	7.23%	99.95%	84.36%	96.70%	13.31%
VGG 11—0	35.53%	99.62%	77.41%	97.37%	48.70%
VGG 11—10	17.26%	99.80%	76.14%	96.91%	28.14%
VGG 11—20	7.56%	99.86%	66.07%	96.62%	13.57%
VGG 11—30	2.38%	99.90%	47.26%	96.48%	4.53%
VGG 11—40	6.45%	99.55%	34.05%	96.28%	10.84%
VGG 11—50	2.51%	99.95%	64.21%	96.53%	4.82%
AlexNet—0	37.93%	99.54%	74.85%	97.38%	50.34%
AlexNet—10	21.08%	99.80%	79.65%	97.04%	33.34%
AlexNet—20	12.83%	99.89%	80.65%	96.83%	22.13%
AlexNet—30	8.60%	99.88%	72.91%	96.68%	15.38%
AlexNet—40	0.70%	99.99%	83.97%	96.51%	1.38%
AlexNet—50	2.79%	99.96%	73.63%	96.55%	5.37%

* Refer to Appendix A for the standard deviations of the evaluation metrics for the training results.

**Table 2:** Evaluation indices of different CNN backbone models for crack diagnosis from the MTSS dataset with MB *.

Model—Blur	Evaluation Index				
	Recall	Selectivity	Precision	Accuracy	F1-Score
ResNet 18—0	92.46%	99.19%	85.74%	98.85%	88.97%
ResNet 18—10	54.14%	99.43%	83.32%	97.16%	65.63%
ResNet 18—20	29.59%	99.66%	82.05%	96.14%	43.49%
ResNet 18—30	11.89%	99.85%	80.71%	95.44%	20.72%
ResNet 18—40	5.50%	99.91%	76.66%	95.17%	10.27%
ResNet 18—50	1.12%	99.99%	82.25%	95.03%	2.21%
ResNet 34—0	93.41%	99.18%	85.77%	98.89%	89.43%
ResNet 34—10	57.67%	99.35%	82.33%	97.26%	67.83%
ResNet 34—20	24.31%	99.74%	82.93%	95.95%	37.59%
ResNet 34—30	12.39%	99.85%	81.62%	95.46%	21.52%
ResNet 34—40	5.39%	99.92%	77.93%	95.18%	10.08%
ResNet 34—50	2.29%	99.96%	76.44%	95.06%	4.45%
VGG 11—0	71.36%	98.61%	73.10%	97.25%	72.22%
VGG 11—10	31.12%	99.42%	73.80%	95.99%	43.78%
VGG 11—20	12.47%	99.64%	64.54%	95.26%	20.90%
VGG 11—30	6.70%	99.82%	66.39%	95.15%	12.17%
VGG 11—40	3.74%	99.87%	60.94%	95.05%	7.05%
VGG 11—50	0.47%	99.99%	68.51%	95.00%	0.93%
AlexNet—0	47.05%	99.10%	73.37%	96.49%	57.33%
AlexNet—10	25.27%	99.37%	67.86%	95.65%	36.82%
AlexNet—20	8.40%	99.87%	77.16%	95.28%	15.15%
AlexNet—30	2.83%	99.96%	77.91%	95.08%	5.46%
AlexNet—40	1.96%	99.94%	63.49%	95.02%	3.80%
AlexNet—50	0.88%	99.98%	71.24%	95.01%	1.74%

* Refer to Appendix A for the standard deviations of the evaluation metrics for the training results.

**Table 3:** Evaluation results of ResNet34 on the Kaggle dataset.

Kaggle Dataset	F1-Score	BRISQUE	NIQE	PIQE	CPBD
**ResNet 34—0**	71.25%	33.65	6.42	21.04	0.73
**ResNet 34—10**	62.23%	43.84	7.35	59.70	0.24
**ResNet 34—20**	49.27%	44.39	7.65	73.87	0.23
**ResNet 34—30**	37.51%	44.43	7.85	78.56	0.19
**ResNet 34—40**	27.75%	44.42	8.00	80.92	0.15
**ResNet 34—50**	13.31%	44.37	8.09	82.34	0.11

**Table 4:** Evaluation results of ResNet34 on the MTSS dataset.

MTSS Dataset	F1-Score	BRISQUE	NIQE	PIQE	CPBD
**ResNet 34—0**	89.43%	33.87	3.82	13.77	0.87
**ResNet 34—10**	67.83%	43.42	7.03	69.35	0.12
**ResNet 34—20**	37.59%	43.73	7.47	79.76	0.104
**ResNet 34—30**	21.52%	43.91	7.68	85.53	0.05
**ResNet 34—40**	10.08%	43.84	7.87	85.50	0.00
**ResNet 34—50**	4.45%	43.81	8.06	86.49	0.00

**Table 5:** Histograms of CPBD and PIQE in the Kaggle dataset *.

NR-IQA	CPBD	PIQE
Histogram	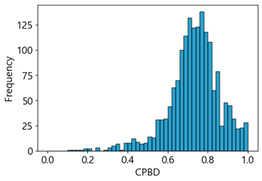	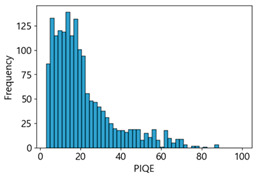
Max	1.0	86.9515
Min	0.1047	2.9123
Mean	0.7322	21.0473
Standard deviation	0.1289	15.4365

* Refer to Appendix B for histograms of PIQE and CPBD as a function of motion-blur (MB) intensity.

**Table 6:** Histograms of CPBD and PIQE in the MTSS dataset *.

NR-IQA	CPBD	PIQE
Histogram	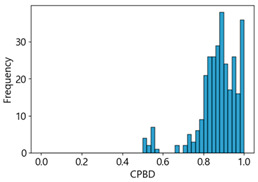	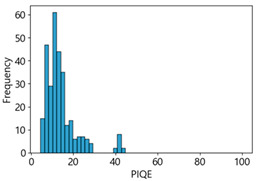
Maximum	1.0	43.1943
Minimum	0.511	4.4171
Mean	0.8707	13.7734
Standard deviation	0.1009	7.6322

* Refer to Appendix B for histograms of PIQE and CPBD as a function of motion-blur (MB) intensity.

**Table 7:** Quality scale and respective score range of PIQE.

Quality Scale	Score Range	Description
Excellent	[0, 20]	The image exhibits minimal perceptual degradation with high clarity and sharpness. Distortions or artifacts do not exist, making it highly suitable for detailed analysis and machine learning applications.
Good	[21, 35]	The image maintains good visual quality with only minor perceptual distortions. Slight blurring or noise may be present, but the structural details remain largely intact, ensuring usability in most practical scenarios.
Fair	[36, 50]	Moderate degradation in image quality is observed. Blurring, noise, or compression artifacts are more prominent, potentially impacting fine details. While still usable, the image may require preprocessing to enhance quality.
Poor	[51, 80]	Significant quality degradation is evident. The image suffers from noticeable blur, noise, or distortions, leading to a substantial loss of detail. Its usability for precise tasks, such as deep learning-based crack detection, is considerably reduced.
Bad	[81, 100]	The image quality is severely degraded, with extreme blurring, noise, or compression artifacts. Structural information is highly compromised, making it unsuitable for analytical or automated processing tasks.

**Table 8:** Quality scale and respective score range of assumed CPBD.

Quality Scale	Score Range
Excellent	[1, 0.8]
Good	[0.81, 0.65]
Fair	[0.64, 0.5]
Poor	[0.51, 0.2]
Bad	[0.21, 0]

**Table 9:** Histograms of threshold ranges for CPBD and PIQE on the Kaggle dataset.

NR-IQA	CPBD	PIQE
Excellent	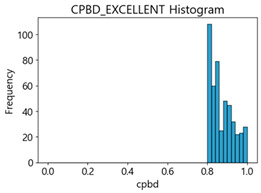	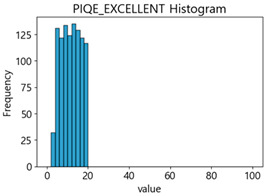
Good	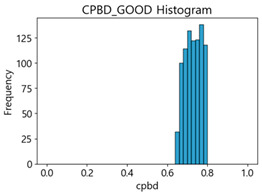	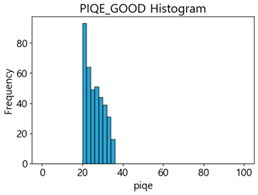
Excellent + Good	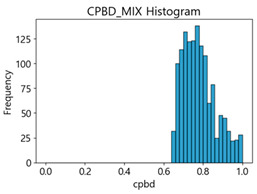	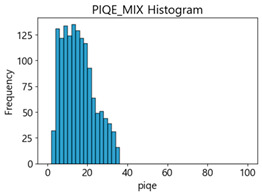
All	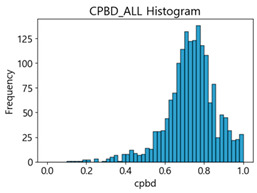	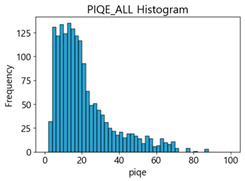

**Table 10:** Histograms of threshold ranges for CPBD and PIQE on the MTSS dataset.

NR-IQA	CPBD	PIQE
Excellent	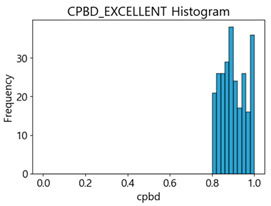	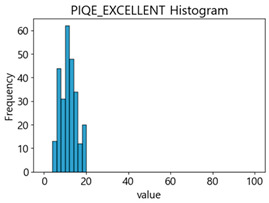
Good	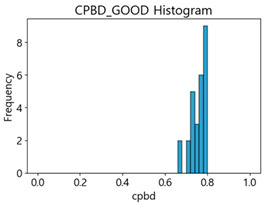	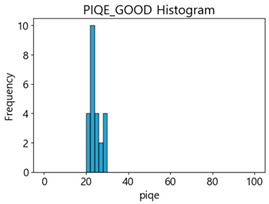
Excellent + Good	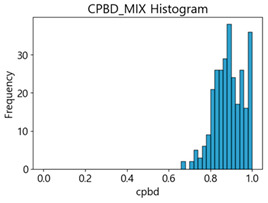	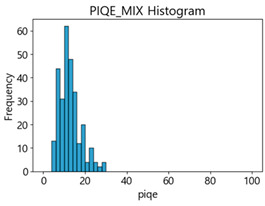
All	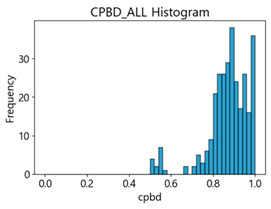	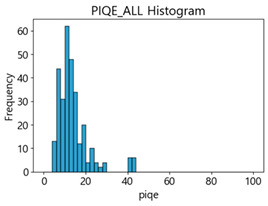

**Table 11:** F1-score results of CNN models with threshold ranges in the Kaggle dataset.

NR-IQA	CNN	Excellent	Good	Excellent + Good	All
CPBD	ResNet18	71.56%	65.95%	70.29%	71.83%
	ResNet34	70.60%	65.03%	69.28%	70.80%
	VGG11	57.21%	53.89%	56.44%	56.88%
	AlexNet	55.59%	47.08%	53.69%	55.46%
PIQE	ResNet18	75.37%	71.15%	72.70%	71.83%
	ResNet34	75.4%	70.01%	71.67%	70.80%
	VGG11	59.40%	57.53%	58.22%	56.88%
	AlexNet	60.06%	55.0%	56.89%	55.46%

**Table 12:** F1-score results of CNN models with threshold ranges in the MTSS dataset.

NR-IQA	CNN	Excellent	Good	Excellent + Good	All
CPBD	ResNet18	87.16%	84.79%	87.03%	86.97%
	ResNet34	87.12%	84.56%	86.68%	86.95%
	VGG11	77.33%	67.11%	76.81%	76.25%
	AlexNet	64.70%	48.21%	63.85%	63.24%
PIQE	ResNet18	87.04%	86.74%	87.03%	86.97%
	ResNet34	86.98%	86.99%	86.98%	86.95%
	VGG11	77.28%	65.44%	76.80%	76.25%
	AlexNet	64.80%	36.05%	63.83%	63.24%

## Data Availability

The original contributions presented in this study are included in the article. Further inquiries can be directed to the corresponding author.

## Appendix A

### Appendix A.1. Standard Deviations of Evaluation Metrics for the CNN Training Results (Kaggle)

**Model—Blur**	**Evaluation Index**				
	**Recall**	**Selectivity**	**Precision**	**Accuracy**	**F1 Score**
ResNet 18—0	0.2892	0.0069	0.3092	0.0189	0.2797
ResNet 18—10	0.2977	0.0076	0.3085	0.0192	0.284
ResNet 18—20	0.302	0.0082	0.307	0.0198	0.285
ResNet 18—30	0.1739	0.0112	0.2096	0.0369	0.1482
ResNet 18—40	0.1138	0.0044	0.2012	0.0374	0.1155
ResNet 18—50	0.0664	0.0043	0.1677	0.0375	0.0726
ResNet 34—0	0.2911	0.0074	0.3027	0.019	0.2786
ResNet 34—10	0.2997	0.0074	0.3027	0.0189	0.2831
ResNet 34—20	0.2992	0.0076	0.3084	0.0197	0.2858
ResNet 34—30	0.3025	0.0081	0.3058	0.0197	0.286
ResNet 34—40	0.2982	0.0081	0.3123	0.0202	0.2865
ResNet 34—50	0.306	0.0083	0.3082	0.0201	0.2888
VGG 11—0	0.3066	0.012	0.3725	0.0259	0.2927
VGG 11—10	0.2754	0.0083	0.3872	0.0269	0.2824
VGG 11—20	0.2773	0.0109	0.3738	0.0268	0.2779
VGG 11—30	0.2729	0.011	0.371	0.0268	0.2744
VGG 11—40	0.2679	0.0115	0.3638	0.0264	0.2686
VGG 11—50	0.2926	0.0163	0.3253	0.0274	0.2685
AlexNet—0	0.2785	0.0088	0.3767	0.0269	0.2828
AlexNet—10	0.3019	0.0108	0.3739	0.026	0.2964
AlexNet—20	0.3017	0.0111	0.3793	0.0254	0.2966
AlexNet—30	0.3046	0.0125	0.3604	0.0254	0.2894
AlexNet—40	0.2633	0.0089	0.373	0.0262	0.2691
AlexNet—50	0.267	0.0104	0.3641	0.0264	0.2685

### Appendix A.2. Standard Deviations of Evaluation Metrics for the CNN Training Results (MTSS)

**Model—Blur**	**Evaluation Index**				
	**Recall**	**Selectivity**	**Precision**	**Accuracy**	**F1 Score**
ResNet 18—0	0.0595	0.0053	0.0284	0.008	0.0393
ResNet 18—10	0.1181	0.0052	0.0659	0.0091	0.0909
ResNet 18—20	0.1189	0.0052	0.0484	0.0102	0.0902
ResNet 18—30	0.1746	0.005	0.0936	0.0121	0.1437
ResNet 18—40	0.1384	0.005	0.0706	0.0102	0.1138
ResNet 18—50	0.1673	0.0049	0.1194	0.0126	0.1415
ResNet 34—0	0.0611	0.0051	0.0324	0.0083	0.0419
ResNet 34—10	0.1876	0.0053	0.1032	0.0119	0.1565
ResNet 34—20	0.1853	0.0053	0.104	0.0128	0.1558
ResNet 34—30	0.1043	0.0052	0.0504	0.0084	0.0768
ResNet 34—40	0.1824	0.0052	0.1213	0.0132	0.1546
ResNet 34—50	0.1786	0.0052	0.111	0.013	0.1486
VGG 11—0	0.1587	0.0098	0.0762	0.0188	0.12
VGG 11—10	0.1734	0.0097	0.0845	0.0187	0.1339
VGG 11—20	0.1807	0.0078	0.0965	0.0183	0.1523
VGG 11—30	0.2031	0.0095	0.1223	0.0233	0.1739
VGG 11—40	0.1956	0.009	0.1161	0.0232	0.1667
VGG 11—50	0.1949	0.0084	0.1122	0.024	0.1668
AlexNet—0	0.2467	0.0146	0.2568	0.0257	0.2366
AlexNet—10	0.2334	0.011	0.2946	0.0251	0.2394
AlexNet—20	0.2187	0.0102	0.3259	0.0265	0.2364
AlexNet—30	0.1929	0.0091	0.3419	0.0276	0.2147
AlexNet—40	0.1729	0.0089	0.3299	0.0286	0.1982
AlexNet—50	0.1724	0.008	0.3212	0.0283	0.1992

## Appendix B

### Appendix B.1. Histograms of NR-IQA in the Kaggle Dataset

**Blur**	**BIRSQ**	**NIQE**
0 (Original)		
10		
20		
30		
40		
50		

### Appendix B.2. Histograms of NR-IQA in the Kaggle Dataset (Continued)

**Blur**	**CPBD**	**PIQE**
0 (Original)		
10		
20		
30		
40		
50		

### Appendix B.3. Histograms of NR-IQA in the MTSS Dataset

**Blur**	**BIRSQUE**	**NIQE**
0 (Original)		
10		
20		
30		
40		
50		

### Appendix B.4. Histograms of NR-IQA in the MTSS Dataset (Continued)

**Blur**	**CPBD**	**PIQE**
0 (Original)		
10		
20		
30		
40		
50		
